# Predictors of podiatry utilisation in Australia: the North West Adelaide Health Study

**DOI:** 10.1186/1757-1146-1-8

**Published:** 2008-08-19

**Authors:** Hylton B Menz, Tiffany K Gill, Anne W Taylor, Catherine L Hill

**Affiliations:** 1 Musculoskeletal Research Centre, Faculty of Health Sciences, La Trobe University, Bundoora, Victoria 3086, Australia; 2 Population Research and Outcome Studies Unit, Department of Health, Adelaide, South Australia, 5000, Australia; 3 Rheumatology Unit, Queen Elizabeth Hospital, Woodville, South Australia, 5011, Australia

## Abstract

**Background:**

Foot problems are highly prevalent in the community; however no large population-based studies have examined the characteristics of those who do and do not access podiatry services in Australia. The aim of this study was to explore patterns of podiatry utilisation in a population-based sample of people aged 18 years and over living in the northwest region of Adelaide, South Australia.

**Methods:**

The North West Adelaide Health Study is a representative longitudinal cohort study of 4,060 people randomly selected and recruited by telephone interview. The interview included questions regarding healthcare service utilisation in the past year. Data were also collected on education, income and major medical conditions.

**Results:**

Overall, 9.5% of the total sample and 17.7% of those who reported foot pain had attended a podiatrist in the past year. Participants who had accessed podiatry treatment were more likely to be female, be aged over 45 years, be obese, and have major chronic medical conditions (osteoporosis, osteoarthritis, diabetes, cardiovascular disease and high blood pressure). Those who reported foot pain but had not accessed a podiatrist were more likely to be male and be aged 20 to 34 years.

**Conclusion:**

Only a small proportion of people who report foot pain have accessed podiatry services in the past year. There is a need to further promote podiatry services to the general community, particularly to men and younger people.

## Background

Foot pain is a highly prevalent condition reported by at least one in five people in the general population [1,2]. The prevalence of foot pain increases with age [1-5], is more prevalent in females [1,2,6,7] and the obese [2,8,9], and is associated with self-reported disability [10], inability to perform activities of daily living [7,11,12] and reduced health-related quality of life [2,13,14]. Despite the significant impact of foot disorders, several intervention studies have shown that foot pain associated with common conditions such as corns and calluses, nail disorders and plantar fasciitis can be effectively managed with a range of conservative and surgical techniques [15,16].

The provision of foot health services to manage foot pain and disability is primarily (although not exclusively) the domain of the podiatry profession [17]. However, although several studies have evaluated foot health service provision in specialist disciplines (such as rheumatology [18-20], diabetes [21,22] and geriatrics [23-27]), few have examined the characteristics of people who do and do not access podiatry services in the general community. In the UK, a population-based survey of 792 people aged over 60 years reported that only 33% of those with foot problems had received podiatry treatment, with those who accessed podiatry being more likely to be female, older, and living alone [28]. Similarly, the Cheshire Foot Pain and Disability Survey of 3,417 people aged between 18 and 80 years reported that only 36% of those with disabling foot pain had accessed podiatry services in the last six months [1], which was partly attributed to a relative shortage of National Health Service podiatry resources for younger people.

In Australia, the 2004–2005 National Health Survey of 25,906 people included a question regarding consultations with health professionals in the last two weeks, and found that 6.7% of the population had consulted a podiatrist [29]. The likelihood of accessing podiatry services increased steadily with age, and those who consulted a podiatrist were twice as likely to be female. However, whether this level of access of podiatry services is adequate cannot be ascertained from these data, as the survey did not collect any information regarding the presence of foot problems. Furthermore, although data were collected on other demographic factors and major health conditions, no analyses were undertaken to compare the characteristics of those who did and did not consult a podiatrist in the last two weeks.

To the authors' knowledge, the only population-based Australian study to examine rates of podiatry utilisation in relation to need was conducted in rural south-east Queensland in 1995 [5]. This study – the West Moreton Rural Health Needs Assessment survey – involved an interviewer-administered general health survey of 600 people aged 18 years and over, and incorporated several questions regarding foot problems and health service utilisation. Of the 154 people who reported foot problems, 102 (66%) had sought treatment in the previous year. However, only 16% reported receiving treatment from a podiatrist, with the majority (71%) seeking treatment from their general practitioner. Consistent with the findings of the National Health Survey [29], women and those aged over 65 years of age were more likely to seek podiatric treatment for their foot problem.

Developing a more thorough understanding of the number and characteristics of people who do and do not consult podiatrists may assist in evaluating the adequacy of podiatry resources and help identify specific gaps in service provision. Therefore, the aim of our study was to explore patterns of podiatry utilisation in those who took part in the North West Adelaide Health Study, a population-based survey of 4,060 people aged 18 years and over living in the northwest region of Adelaide, South Australia.

## Methods

### Setting and study population

The North West Adelaide Health Study (NWAHS) was established in 2000 in the north-western region of Adelaide, South Australia [30]. The north-west region of Adelaide comprises approximately half of the population of the city of Adelaide and a third of the population of the state of South Australia. The region also reflects the demographic profile of the state, covering a broad range of ages and socioeconomic areas. The study was designed in response to a need to assess the prevalence of priority conditions and examine their progression over time in a population-based community-dwelling cohort, to inform policy decisions about health care provision in South Australia.

Participants for Stage 1 of the study (which was conducted between 2000 and July 2003) were recruited randomly from the Electronic White Pages telephone listings and an initial telephone interview was conducted. The overall response rate for an interview and attendance at the clinic assessment was 49.4%. Those within each household, who were last to have a birthday and aged 18 years and over were interviewed and invited to attend a clinic assessment.

Between 2004 and 2006, Stage 2 of the study was conducted. Where possible, all participants were contacted and invited to participate in a Computer Assisted Telephone interview (CATI), a self-completed questionnaire and/or a clinic assessment. Stage 2 specifically focused on the collection of information relating to musculoskeletal conditions, with n = 3,502 respondents providing information relating to podiatry use in the telephone questionnaire and n = 3,206 respondents attending the clinic. Ethical approval for the study was obtained from the North West Adelaide Health Service Ethics of Human Research Committee, and written informed consent was obtained from all participants.

### Data collection

As part of the self completed questionnaire, information relating to demographics, smoking, self-reported prevalence of diabetes and levels of physical activity using the questions from the Australian National Health Survey [31] were collected. As part of the clinic assessment, height, weight, waist and hip circumference were measured, blood was taken, and all participants attending the clinic in Stage 2 were asked: "On most days do you have pain, aching or stiffness in either of your feet?". If they answered yes to this question, they were regarded to have foot pain. As part of the CATI, the self-reported prevalence of osteoporosis and cardiovascular disease were determined, as was the health service utilisation in the past 12 months.

### Statistical analysis

Data were weighted by age and sex, and probability of selection within the household, to the population of the north-west suburbs of Adelaide. Analysis was undertaken using SPSS Version 15 to determine the prevalence of podiatry consultation, and associations between age, sex, body mass index (BMI), selected chronic diseases, health risk factors and musculoskeletal pain (including foot pain). Frequencies were determined for the prevalence values (foot pain and podiatry use) and demographic characteristics of the sample. Univariate and multivariate logistic regression analyses were also undertaken. Variables that were significant at *p *< 0.25 at a univariate level were included in the logistic regression models as described by Hosmer and Lemeshow [32]. The multivariate logistic regression used a backwards stepwise method, with non-significant variables removed at each step. The Hosmer and Lemeshow Goodness of Fit test is an indicator of the fit of the model; a significant value indicates that the model is not a good fit for the data [32]. The Omnibus test also is a test of how well the independent variables in the model jointly predict the dependent variable. If significant, this indicates that the model is a good fit for the data [33]. Variables in the final model were significant at the level of *p *< 0.05.

## Results

### Sample characteristics

Sample characteristics are shown in Table 1. The characteristics of the NWAHS cohort demonstrate that this is a relatively young, heavy cohort with 38% under 40 years; and the mean BMI in the overweight range. A previous analysis of this dataset indicated that 17% had foot pain, with those reporting foot pain more likely to be female, be aged 50 years and over, be obese and also report knee, hip and back pain [2].

**Table 1:** Demographic characteristics of participants in the NWAHS.

Variable	
Sex	
Male	1718 (49.1)
Female	1784 (50.9)
Age	
20 to 34 years	996 (28.4)
35 to 44 years	711 (20.3)
45 to 54 years	620 (17.7)
55 to 64 years	477 (13.6)
65 to 74 years	355 (10.1)
75 years and over	344 (9.8)
Education	
Secondary	1450 (44.5)
Trade/Apprentice/Certificate/Diploma	1202 (36.9)
Degree	560 (17.2)
Other/not stated	49 (1.5)
Income	
Up to $20,000	613 (18.8)
$20,000–$40,000	745 (22.8)
$40,001–$60,000	697 (21.4)
$60,001–$80,000	495 (15.2)
$80,001–$100,000	314 (9.6)
More than $100,000	263 (8.1)
Not stated	133 (4.1)

Note: Values are n (%) unless otherwise noted.

### Prevalence and predictors of podiatry service utilisation

Overall, n = 334 (9.5%; 95% CI 8.6–10.5) respondents who responded to questions regarding health service use reported that they had consulted a podiatrist in the last 12 months. Participants who had accessed podiatry treatment were more likely to be female, be aged over 45 years, have completed only secondary education, earn $20,000 or less per year, be obese, have major chronic medical conditions (osteoporosis, osteoarthritis, diabetes, cardiovascular disease and high blood pressure), were less likely to consume intermediate to high risk levels of alcohol, or be current smokers (*p *< 0.05, unadjusted univariate analysis) (Table 2). Sex, age, arthritis, diabetes and smoking status were confirmed as significant predictors in multivariate logistic regression (Hosmer and Lemeshow Goodness of Fit χ^2 ^= 10.96, *p *= 0.204; Omnibus test χ^2 ^= 295.93, *df *= 9, *p *< 0.001) (Table 3).

**Table 2:** Characteristics of participants who accessed podiatry services (n = 334) in the previous 12 months (univariate analysis).

Variable	n	%	Odds ratio (95% CI)	*p *value
Sex				
Male	102	5.9	1.00	
Female	232	13.0	2.38 (1.86–3.03)	< 0.001
Age				
20 to 34 years	37	3.7	1.00	
35 to 44 years	35	4.9	1.36 (0.85–2.18)	0.206
45 to 54 years	39	6.3	1.75 (1.11–2.78)	0.017
55 to 64 years	52	10.9	3.20 (2.07–4.96)	< 0.001
65 to 74 years	60	17.0	5.35 (3.48–8.22)	< 0.001
75 years and over	110	32.1	12.32 (8.26–18.37)	< 0.001
Education				
Secondary	167	11.8	1.00	
Trade/Apprentice/Certificate/Diploma	101	8.5	0.70 (0.54–0.91)	0.007
Degree or higher	36	6.4	0.51 (0.35–0.75)	< 0.001
Other/not stated	5	10.5	0.88 (0.34–2.25)	0.787
Income				
Up to $20,000	94	15.9	1.00	
$20,001–$40,000	80	11.0	0.65 (0.47–0.90)	0.008
$40,001–$60,000	51	7.4	0.42 (0.30–0.61)	< 0.001
$60,001–$80,000	32	6.5	0.37 (0.24–0.56)	< 0.001
$80,001–$100,000	14	4.6	0.26 (0.14–0.45)	< 0.001
More than $100,000	20	7.4	0.42 (0.25–0.71)	0.001
Not stated	17	13.7	0.84 (0.48–1.45)	0.524
Chronic conditions				
Osteoporosis	37	28.0	4.04 (2.72–6.01)	< 0.001
Arthritis	147	19.8	3.48 (2.75–4.40)	< 0.001
Diabetes	65	29.4	4.58 (3.34–6.30)	< 0.001
Cardiovascular disease	55	23.6	3.32 (2.40–4.60)	< 0.001
High blood pressure	116	14.3	1.85 (1.45–2.37)	< 0.001
High cholesterol	126	9.9	1.01 (0.80–1.29)	0.924
Obese (BMI ≥ 30 kg/m^2^)	108	11.6	1.31 (1.03–1.68)	0.031
Alcohol consumption				
Non drinker (no risk)	159	10.2	1.00	
Low risk	129	9.7	0.95 (0.74–1.21)	0.655
Intermediate to very high risk	7	4.1	0.38 (0.18–0.81)	0.013
Current smoker	24	3.7	0.31 (0.20–0.48)	< 0.001
No physical activity (sedentary)	79	9.5	0.99 (0.76–1.31)	0.967

Note: The weighting of data can result in rounding discrepancies or totals not adding.

**Table 3:** Multivariate predictors of accessing podiatry services in the last 12 months.

Variable	Odds ratio (95% CI)	*p *value
Sex		
Male	1.00	
Female	2.32 (1.77–3.05)	< 0.001
Age		
20 to 34 years	1.00	
35 to 44 years	1.21 (0.75–1.94)	0.439
45 to 54 years	1.16 (0.71–1.89)	0.554
55 to 64 years	1.75 (1.09–2.82)	0.021
65 to 74 years	2.53 (1.54–6.11)	< 0.001
75 years and over	5.99 (3.81–9.42)	< 0.001
Chronic conditions		
Arthritis	1.65 (1.23–2.22)	0.001
Diabetes	3.11 (2.17–4.46)	< 0.001
Current smoker	0.46 (0.29–0.73)	0.001

Of those who attended the clinic assessment and reported that they had foot pain (n = 538), 17.7% (95% CI 14.7–21.2, n = 95) had consulted a podiatrist in the last 12 months. Those who reported foot pain but had not accessed a podiatrist were more likely to be male, be aged 20 to 34 years, earn between $40,000 and $60,000 per year and be current smokers, or were less likely to have major chronic medical conditions (osteoporosis, osteoarthritis, diabetes, cardiovascular disease and high blood pressure) (*p *< 0.05, unadjusted univariate analysis) (Table 4). Sex and age were confirmed as significant predictors in multivariate logistic regression analyses (Hosmer and Lemeshow Goodness of Fit χ^2 ^= 4.14, *p *= 0.844; Omnibus test χ^2 ^= 53.57, *df *= 6, *p *< 0.001) (Table 5).

**Table 4:** Characteristics of participants who reported foot problems and did not access podiatry services (n = 443) in the last 12 months (univariate analysis).

Variable	n	%	Odds ratio (95% CI)	*p *value
Sex				
Female	233	77.2	1.00	
Male	210	88.9	2.36 (1.45–3.84)	0.001
Age				
75 years and over	40	58.7	1.00	
65 to 74 years	61	73.4	1.94 (0.98–3.84)	0.058
55 to 64 years	87	84.0	3.69 (1.81–7.52)	< 0.001
45 to 54 years	105	86.0	4.32 (2.14–8.74)	< 0.001
35 to 44 years	62	89.3	5.85 (2.38–14.37)	< 0.001
20 to 34 years	88	95.9	16.43 (5.26–51.28)	< 0.001
Education				
Secondary	249	82.4	1.00	
Trade/Apprentice/Certificate/Diploma	142	81.9	0.96 (0.59–1.57)	0.883
Degree or higher	39	82.0	0.97 (0.44–2.16)	0.940
Other/not stated	12	84.1	1.12 (0.26–4.91)	0.877
Income				
Up to $20,000	90	72.0	1.00	
$20,001–$40,000	121	81.8	1.74 (0.98–3.09)	0.057
$40,001–$60,000	92	91.0	3.91 (1.78–8.58)	0.001
$60,001–$80,000	43	78.8	1.45 (0.68–3.09)	0.342
$80,001–$100,000	33	85.4	2.28 (0.86–6.04)	0.097
More than $100,000	35	91.3	4.09 (1.23–13.52)	0.021
Not stated	28	88.5	2.98 (0.93–9.50)	0.065
Chronic conditions				
Osteoporosis	24	64.5	0.36 (0.18–0.72)	0.004
Arthritis	173	75.3	0.42 (0.26–0.66)	< 0.001
Diabetes	42	68.7	0.42 (0.23–0.76)	0.004
Cardiovascular disease	36	68.4	0.42 (0.22–0.79)	0.007
High blood pressure	134	76.4	0.57 (0.36–0.90)	0.015
High cholesterol	186	80.3	0.76 (0.49–1.19)	0.235
Obese (BMI ≥ 30 kg/m^2^)	187	81.9	0.84 (0.61–1.49)	0.841
Alcohol consumption				
Non drinker (no risk)	198	80.5	1.00	
Low risk	198	82.8	1.17 (0.74–1.85)	0.508
Intermediate to very high risk	29	95.1	4.68 (0.89–24.76)	0.068
Current smoker	90	92.6	3.14 (1.42–6.93)	0.005
No physical activity (sedentary)	136	83.5	1.10 (0.67–1.82)	0.707

Note: The weighting of data can result in rounding discrepancies or totals not adding.

**Table 5:** Multivariate predictors of not accessing podiatry services in the last 12 months in people who reported foot pain.

Variable	Odds ratio (95% CI)	*p *value
Sex		
Female	1.00	
Male	2.11 (1.27–3.50)	0.004
Age		
75 years and over	1.00	
65 to 74 years	1.94 (0.97–3.88)	0.061
55 to 64 years	3.53 (1.72–7.26)	0.001
45 to 54 years	4.26 (2.09–8.67)	< 0.001
35 to 44 years	5.42 (2.19–13.42)	< 0.001
20 to 34 years	15.08 (4.80–47.34)	< 0.001

## Discussion

The aim of this study was to examine the prevalence and predictors of podiatry service utilisation in a population-based sample of people aged 18 years and over who took part in the North West Adelaide Health Study (NWAHS). The findings indicate that 9.5% of the cohort had consulted a podiatrist in the past 12 months. Of those who reported foot pain, only 17.7% had consulted a podiatrist. Our analysis indicated that the typical podiatry patient is an older, obese woman with limited education, relatively low income, and multiple chronic diseases. In contrast, those with foot problems who have not consulted a podiatrist tended to be younger men without chronic diseases.

The total proportion of people who reported accessing podiatry services in the NWAHS (9.5%) was higher than the 2004–2005 Australian National Health Survey (6.7%) [31] and the West Moreton Rural Health Needs Assessment survey (3%) [5]. The difference between the current study and the National Health Survey is most likely due to the different timeframes contained within the health care utilisation questionnaires used (previous 12 months for the NWAHS compared to the previous two weeks in the National Health Survey). However, it is also possible that the NWAHS population had greater access to podiatry than the national average. The Australian Institute for Health and Welfare's Podiatry Labour Force study estimated that in 2003, the number of full-time equivalent podiatrists per 100,000 population in South Australia was 17.4, higher than all other states included in the survey (Victoria: 13.0, Tasmania: 12.4, New South Wales: 9.3 and Queensland: 7.7) [34].

The proportion of people who reported foot pain and who had consulted a podiatrist (17%) was substantially lower than similar studies conducted in the UK (33 to 36%) [1,28], but similar to the rate reported in the West Moreton Rural Health Needs Assessment survey (16%) [5]. In the UK, the National Health Service provides free podiatry care to approximately 4% of the population, with the majority of recipients being aged over 65 years [35]. In Australia, relatively limited podiatry services are provided by the public sector, and in most settings access to podiatry is restricted to those with "high risk" feet, i.e.: those with chronic conditions such as diabetes or rheumatoid arthritis. Subsequently, the awareness and utilisation of podiatry among older people is likely to be higher than younger people [27]. The lack of publicly-funded podiatry services for people without chronic diseases, combined with an inability or reluctance to pay for private services, may explain the very low levels of podiatry consultation in younger people (as low as 4 to 10% in those aged 20 to 44 years).

However, it is also possible that some degree of foot health service provision is currently being met by other health care professionals, particularly general practitioners. A survey of 1,130 people aged over 65 years of age in the Netherlands indicated that 30% sought foot treatment from their general practitioner rather than a podiatrist/chiropodist [26]. Similarly, in the West Moreton Rural Health Needs Assessment survey, 71% of those with a foot problem had consulted their general practitioner, with no podiatry consultations reported by those aged 18 to 24 years [5]. Interestingly, the National Health Interview Survey in the US indicated that while treatment of corns, calluses and nail disorders were almost exclusively provided by podiatrists, management of musculoskeletal foot conditions and acute injuries (such as ankle sprains) were more likely to be managed by medical practitioners [3]. Given the high prevalence of older people accessing podiatry services, it is possible that younger people do not consider consulting a podiatrist for musculoskeletal foot conditions, as they associate podiatry with routine management of skin and nail problems in older people. If this is correct, there may be a need for the podiatry profession to promote a greater awareness of the scope of podiatry practice to young and middle-aged people.

Consistent with anecdotal observations, our results indicate that the typical patient attending podiatry is an older, obese woman with limited education, relatively low income, and multiple chronic diseases. This patient profile is not surprising given the available evidence relating to the role of increased age [1-5], female sex [1,2,6,7], obesity [2,5,8,9] and comorbidities [2,9,12,36] in the development of foot problems. The role of socio-economic status, however, is equivocal. Lower levels of education have been found to be associated with foot problems in some studies [3,9] but not others [12,13,36]. Similarly, while some studies have found that people with foot problems have lower income levels [3] others have failed to find such an association [9,13]. These discrepancies are likely to reflect differences in how income levels are defined, differences in educational systems between countries, and variability in adjustment for confounders in the statistical models. Nevertheless, in the current study the association between accessing podiatry services and socio-economic factors was no longer significant after other variables were considered.

The major strength of this study is the use of a population-based sample with excellent response rates over a broad age range. However, the findings of this study need to be interpreted in the context of several limitations. Firstly, we defined foot pain according to a single question rather than using foot-specific questionnaires, such as the Manchester Foot Pain and Disability Index [14,37] or Foot Health Status Questionnaire [38]. Secondly, we were unable to examine the participants' feet in the study to ascertain the underlying cause of their pain. Thirdly, we did not ask participants whether they had accessed other health care professionals for management of their foot pain. As such, we cannot necessarily conclude that the proportion of people with foot pain who have not accessed a podiatrist is an accurate indicator of unmet need.

Despite these limitations, the results of this study provide the first detailed insights into the number and characteristics of people who do and do not access podiatry services in Australia, based on a large representative sample. The findings may assist in the future planning and development of foot health services, and provide some direction for promotional activities for the podiatry profession. Although the important role that podiatry plays in the maintenance of foot health in older people should not be ignored, there would appear to be a large number of young to middle-aged people with foot pain who are currently unaware of, or unable to access, podiatry services in Australia.

## Conclusion

The findings of this population-based study indicate that approximately 10% of the general population has consulted a podiatrist in the past 12 months. Those who attend podiatry are more likely to be female, be aged over 45 years, be obese, and have major chronic medical conditions. The large proportion of people who report foot pain but have not accessed podiatry services (82%) suggests that there may be a need to further promote podiatry services to the general community, particularly to men and younger people.

## Competing interests

HBM is Editor-in-Chief of the *Journal of Foot and Ankle Research*. It is journal policy that editors are removed from the peer review and editorial decision making processes for papers they have co-authored.

## Authors' contributions

AWT, TKG, and CLH conceived the study design, TKG conducted the statistical analysis, HBM and CLH interpreted the results, HBM drafted the manuscript, and all authors read and approved the final manuscript.
